# Effect of Double Substitution in Cationic Chitosan Derivatives on DNA Transfection Efficiency

**DOI:** 10.3390/polym12051057

**Published:** 2020-05-05

**Authors:** Veronika D. Badazhkova, Sergei V. Raik, Dmitry S. Polyakov, Daria N. Poshina, Yury A. Skorik

**Affiliations:** 1 Institute of Macromolecular Compounds of the Russian Academy of Sciences, Bolshoi pr. VO 31, 199004 St. Petersburg, Russia; badazhkova96@mail.ru (V.D.B.); raiksv@gmail.com (S.V.R.); poschin@yandex.ru (D.N.P.); 2 Division of Pharmaceutical Biosciences, Faculty of Pharmacy, University of Helsinki, P.O. Box 56, FI-00014 Helsinki, Finland; 3 Institute of Experimental Medicine, Akademika Pavlova st. 12, 197376 St. Petersburg, Russia; ravendoctor@mail.ru

**Keywords:** chitosan, polyplex, cell transfection, gene delivery

## Abstract

Recently, much effort has been expended on the development of non-viral gene delivery systems based on polyplexes of nucleic acids with various cationic polymers. Natural polysaccharide derivatives are promising carriers due to their low toxicity. In this work, chitosan was chemically modified by a reaction with 4-formyl-*n,n,n*-trimethylanilinium iodide and pyridoxal hydrochloride and subsequent reduction of the imine bond with NaBH_4_. This reaction yielded three novel derivatives, *n*-[4-(*n*’,*n*’,*n*’-trimethylammonium)benzyl]chitosan chloride (TMAB-CS), *n*-[(3-hydroxy-5-(hydroxymethyl)-2-methyl-4-pyridine)methyl]chitosan chloride (Pyr-CS), and *n*-[4-(*n*’,*n*’,*n*’’-trimethylammonium)benzyl]-*n*-[(3-hydroxy-5-(hydroxymethyl)-2-methyl-4-pyridine)methyl]chitosan chloride (PyrTMAB-CS). Their structures and degrees of substitution were established by ^1^H NMR spectroscopy as DS_1_ = 0.22 for TMAB-CS, DS_2_ = 0.28 for Pyr-CS, and DS_1_ = 0.21, DS_2_ = 0.22 for PyrTMAB-CS. Dynamic light scattering measurements revealed that the new polymers formed stable polyplexes with plasmid DNA encoding the green fluorescent protein (pEGFP-N3) and that the particles had the smallest size (110–165 nm) when the polymer:DNA mass ratio was higher than 5:1. Transfection experiments carried out in the HEK293 cell line using the polymer:DNA polyplexes demonstrated that Pyr-CS was a rather poor transfection agent at polymer:DNA mass ratios less than 10:1, but it was still more effective than the TMAB-CS and PyrTMAB-CS derivatives that contained a quaternary ammonium group. By contrast, TMAB-CS and PyrTMAB-CS were substantially more effective than Pyr-CS at higher polymer:DNA mass ratios and showed a maximum efficiency at 200:1 (50%–70% transfected cells). Overall, the results show the possibility of combining substituent effects in a single carrier, thereby increasing its efficacy.

## 1. Introduction

The task of gene therapy or other promising trends in modern medicine is to treat diseases by introducing nucleic acids that can affect gene expression in affected cells [1]. However, an essential part of this task is finding a vector that can efficiently deliver a gene into the target cells [2]. Today, viral and non-viral vectors are undergoing investigation at different stages of clinical and preclinical trials. Viral vectors show high transfection efficiency, but they have some significant shortcomings, including immunogenicity [3,4], insertional mutagenesis [5], and low gene transfer capacity [6,7]. Their use is also further complicated by the complexities involved in industrial virus production. A further complication was exemplified by the famous Gelsinger case in 1999, in which an 18-year-old patient died from an unexpectedly severe immune reaction four days after injection with a modified adenovirus into the liver [4]. These drawbacks have resulted in increased interest in the use of non-viral vectors in gene therapy.

Non-viral delivery systems demonstrate less immunogenicity than viral ones, while also excluding the risk of insertional mutagenesis, affording a higher gene transfer capacity, and offering the possibility of combining different cargos within a single vector [8]. The commercial production of non-viral systems is also safer, simpler and more cost-effective compared to viral systems. Among the available non-viral systems, complexes based on cationic lipids [9] or on natural and synthetic polymers [10] are currently the best studied. The polyplexes based on synthetic polymers have shown a fairly high toxicity; therefore, vectors based on low-toxicity polymers of natural origin are currently of great interest [11]. 

One of those natural polymers is chitosan (CS)—a cationic linear polysaccharide consisting of randomly distributed *d*-glucosamine (>50%) and *n-*acetyl-*d*-glucosamine units. CS is soluble in dilute solutions of organic and mineral acids, has low toxicity, and undergoes chemical modification with relative ease [12]. The amino groups of CS become protonated at acidic pH and gain the ability to bind negatively charged DNA to form nanoscaled polyplexes. These polyplexes are promising delivery vectors because they protect the bound DNA from nuclease degradation and allow its delivery into cells via endocytosis [13,14]. However, chitosan itself has some limitations, including a low solubility in water at physiological pH and a relatively low transfection efficiency [15]. Fortunately, chemical modification can result in a polymer with more desirable properties [16,17]. 

CS can be modified by reactions of its amino groups with aldehydes to form Schiff bases, followed by subsequent reduction with NaBH_4_. The main advantages of this type of modification are the mild reaction conditions (the reactions occur at room temperature in aqueous media) and the formation of a stable product [18]. In our previous studies using this approach, we obtained CS derivatives containing a quaternary ammonium substituent, (*n*,n,*n*-trimethylammonium)benzyl (TMAB), with different degrees of substitution (DS). The obtained derivatives demonstrated good solubility in water and low toxicity; however, their transfection efficiencies were significantly lower than that of the commercially available Lipofectin transfection agent. Nonetheless, further work has confirmed the possibility of controlling the DS by changing the reagent ratios, reaction time, and pH [19].

A few studies have examined the influence of CS substituents on transfection efficiency. For example, we previously showed that the DNA transfection efficiency in Calu-3 cells was significantly better with methylglycol-chitosan than with diethylaminoethyl-chitosan [20]. Similarly, Sajomsang et al. [21] synthesized and characterized CS derivatives containing *n*-(4-*n*,*n*-dimethylaminocinnamyl), *n*-(4-*n*,*n*-dimethylaminobenzyl), and *n*-(4-pyridinylmethyl) moieties and found that *n*-(4-pyridinylmethyl)chitosan chloride was the most promising potential delivery vehicle. The *n*-pyridinium position was subsequently confirmed to impart the high transfection efficiency and low toxicity of that derivative [22]. Self-assembled stearic acid-CS micelles also showed low cell toxicity and high transfection efficiency [23], due to the introduction of a hydrophobic moiety to CS that promoted ready binding to cells and facilitated DNA dissociation inside the cell [24].

In the present work, we synthesized CS derivatives containing either a quaternary ammonium group, pyridine, or both substituents to investigate the effect of substituent structure on the transfection efficiency and buffer capacity. A previously obtained TMAB-CS was used as the derivative with a quaternary ammonium group, as it had shown satisfactory transfection results. The chosen pyridine substituent was pyridoxal, a natural low-toxicity compound and one of the forms of vitamin B6. Other evaluations included polyplex formation with plasmid DNA, morphology of the polyplexes, and the transfection efficiency of polyplexes in the HEK293 cell line.

## 2. Materials and Methods

### 2.1. Materials

CS from crab shells (Bioprogress, Schelkovo, Russia) had the following characteristics: a viscosity average molecular weight of 3.7 × 10^4^ and a degree of acetylation (DA) of 0.26 [19]. We prepared 4-formyl-*n*,n,*n*-trimethylanilinium iodide (FTMA) as previously described [19]. The 4-(*n*,n-dimethylamino)benzaldehyde, pyridoxal hydrochloride, sodium borohydride, and methyl iodide were purchased from Sigma Aldrich (St. Louis, MO, USA).

### 2.2. Synthesis of n-[(3-hydroxy-5-(hydroxymethyl)-2-methyl-4-pyridine)methyl]chitosan Chloride (Pyr-CS)

CS (0.5 g, 2.6 mmol) was dissolved in 50 mL 0.2 M HCl. The pH was adjusted to the desired value by adding NaHCO_3_ and monitoring with a pH meter. Pyridoxal hydrochloride (0.53 g, 2.6 mmol) was added to the CS solution, and the reaction mixture was stirred at room temperature for 3 h. A four-fold excess (with respect to pyridoxal) of dry NaBH_4_ (0.39 g, 10.4 mmol) was then added portionwise. The resulting polymer was precipitated by the addition of 50 mL acetone, separated from the supernatant by centrifugation, and redissolved in 20 mL 0.2 M HCl. The polymer was purified using dialysis membranes with MWCO 12,000–14,000 (Orange Scientific, Braine-l’Alleud, Belgium) for two days against a 1% NaCl solution and then for another three days against distilled water. The purified solutions were freeze-dried in a Freeze Dryer 10N (Shanghai Drawell Scientific Instrument Co., Shanghai, China) at −50 °C in vacuo for 48 h.

### 2.3. Synthesis of n-[4-(n’,n’,n’-trimethylammonium)benzyl]chitosan Chloride (TMAB-CS)

CS (0.5 g, 2.6 mmol) was dissolved in 50 mL 1% acetic acid. The pH was adjusted to the desired value by adding NaHCO_3_ and monitoring with a pH meter. FTMA (0.76 g, 2.6 mmol) was dissolved in 5 mL water and added dropwise to the CS solution under vigorous stirring with a magnetic stirrer. The reaction mixture was then stirred at room temperature for 4 h. A four-fold excess (with respect to FTMA) of dry NaBH_4_ (0.39 g, 10.4 mmol) was then added portionwise. Separation and purification were carried out as described in Section 2.2.

### 2.4. Synthesis of n-[4-(n’,n’,n’-trimethylammonium)benzyl]-N-[(3-hydroxy-5-(hydroxymethyl)-2-methyl-4-pyridine)methyl]chitosan Chloride (PyrTMAB-CS)

CS (0.5 g, 2.6 mmol) was dissolved in 50 mL 0.2 M HCl. The pH was adjusted to the desired value by adding NaHCO_3_ and monitoring with a pH meter. Pyridoxal (0.53 g, 2.6 mmol) and FTMA (0.76 g, 2.6 mmol) were added to the CS solution, and the reaction mixture was stirred at room temperature for 3 h. A four-fold excess (with respect to the aldehydes) of dry NaBH_4_ (0.79 g, 20.8 mmol) was then added portionwise. Separation and purification were carried out as described in Section 2.2. 

### 2.5. Characterization of Polymers

The ^1^H NMR spectra were obtained using a Bruker Avance 400 spectrometer (Bruker, Billerica, MA, USA) at 70 °C with an operating frequency of 400 MHz. Samples were prepared by dissolution of 5 mg of each polymer in 1% CF_3_COOH in D_2_O.

The intrinsic viscosity of CS was determined by viscometry using an Ubbelohde capillary viscometer (Design Bureau Pushchino, Pushchino, Russia) at 20 °C with 0.33 M acetic acid/0.3 M NaCl as solvent. The viscosity-average molecular weight (*M*_η_) of CS was calculated using the Mark–Houwink equation [η] = 3.41 × 10^−3^ × *M*_η_^1.02^ [25]; the intrinsic viscosity [η] = 15.6 dL/g.

### 2.6. Spectrophotometric Measurements and pKa Calculations

A 2.5 mg sample of polymer was dissolved in 2 mL 0.1 M HCl and diluted with H_2_O to 0.125 mg/mL. The pH was then adjusted with 0.5 and 0.1 M NaOH solutions and the UV spectrum was recorded in a range of 220–400 nm with a Shimadzu UV-1700 spectrophotometer (Shimadzu, Kyoto, Japan). TableCurve 2D software (Systat Software Inc., San Jose, CA, USA) was then used to fit the absorbance–pH dependence at a given wavelength with sigmoid functions to determine the p*K*a.

### 2.7. Preparation of Polymer: DNA Polyplexes

The freeze-dried derivatives were dissolved at a concentration of 200 ng/μL in phosphate buffered saline (PBS, pH 7.4) and filtered through a sterilizing filter with a pore diameter of 0.22 μm. The solution of pEGFP-N3 DNA (515 ng/μL) was mixed with the TMAB-CS solution in various mass ratios to give a final DNA concentration of 5 ng/μL. The solution was thoroughly mixed and allowed to stand for 30 min at room temperature to allow the formation of the polyplexes.

### 2.8. Dynamic Light Scattering (DLS)

The hydrodynamic radius (*R*_h_) and ζ-potential of the polyplexes was measured by DLS using a Photocor Compact-Z instrument (Photocor, Moscow, Russia) equipped with a He-Ne laser as a light source (659.7 nm, 25 mW power). The measurements were carried out at 20 °C at a scattering angle of 90°. *R*_h_ was determined by fitting the autocorrelation function using a DynaLS program (Alango Technologies Ltd., Tirat Carmel, Israel) with cumulant expansion. ζ-Potential was calculated from electrophoretic mobility using Henry equation.

### 2.9. Polymer:DNA Binding Assay

The ability of the obtained derivatives to bind DNA was evaluated by a method based on fluorescence quenching of an intercalated dye, which was ethidium bromide (EtBr) in this case. The EtBr was added to phosphate buffered saline (PBS, pH 7.4) and the emission of this solution was used as a reference signal. The solution of EtBr was then titrated with the PEGFP-N3 plasmid DNA solution (2.5 ng/mL) from 0.1 to 10 equivalents of DNA. After each addition of DNA to the EtBr solution, the emission was detected using a Shimadzu RF-5301PC spectrofluorometer (Shimadzu, Kyoto, Japan). The binding ability was calculated as the relative fluorescence change. 

### 2.10. Atomic Force Microscopy (AFM)

The morphology of the polyplexes was determined by atomic force microscopy (AFM) using a Smena scanning probe microscope (NT-MDT, Zelenograd, Russia). The polyplexes were prepared at polymer:DNA ratios of 5 and allowed to stand for 30 min at room temperature. A 5 μL sample of each solution was placed onto freshly cleaved mica and dried at room temperature overnight. All images were obtained in tapping mode in air using a cantilever with curvature radius of 10 nm and a force constant of 60 N/m. The scan rate was 0.8 Hz over a selected area.

### 2.11. Transfection of the HEK293 Cell Line

The HEK293 cells were cultured at 37 °C in a CO_2_ incubator (5% CO_2_, 100% humidity) in DMEM/F12 medium supplemented with 10% fetal-calf serum, 300 μg/mL L-glutamine, 5000 units/mL penicillin G, 50 μg/mL streptomycin, and 25 μg/mL amphotericin B. Confluent cells were disintegrated with EDTA and seeded in 12-well plates with coverslips on the well bottom 24 h before transfection. The number of cells seeded per well allowed achieving 10% confluence on the glass surface in 24 h. 70% of the medium was replaced by fresh medium 4 h before transfection. The HEK293 cells were transfected with the desired volume of polyplex solution. The polyplex solution always contained 500 ng of plasmid DNA per well. pEGFP circular plasmid was isolated from agarose gel with WizardSV Gel and the PCR Clean-Up System (Promega, Madison, WI, USA) according to the manufacturer’s instructions. After 72 h, the transfection efficiency was estimated by counting the numbers of cells expressing the green fluorescent protein.

### 2.12. Statistical Analysis

Statistical analysis was performed using GraphPad Prism software (Graphpad Software LLC, San Diego, CA, USA). Differences between groups were assessed by one-way ANOVA; significance was defined as *p* < 0.05. Post hoc analysis was performed using Dunnett’s multiple comparisons test.

## 3. Results and Discussion 

### 3.1. Synthesis and NMR Characterization of Cationic Chitosan Derivatives

The transfection efficiency of CS is influenced by several factors, such as MW and DA. CS with a low molecular weight (*M*_W_ = 3000–4000) has better solubility and provides a higher level of gene expression compared to high molecular weight CS [26]; however, it is more easily degraded by hydrolytic enzymes and the intestinal bacterial flora [27]. An increase in the molecular weight of CS (*M*_W_ > 1 × 10^5^) increases the stability and DNA binding strength, but this can negatively affect the release of DNA from the polyplex and lead to the formation of aggregates [28,29]. DA affects the order of the structure, the solubility, and the cationic density of the polymer. Therefore, obtaining stable polyplexes capable of transfecting cells required the use of CS with a DA that exceeded 65% [16]. For this reason, crab CS with a molecular weight of 3.7 × 10^4^ and a DA of 0.26 was used in this work.

Polyplexes of CS and DNA can penetrate the cell membrane by endocytosis [30]. Subsequently, an endosome containing the polymer forms inside the cell. The endosome then matures and takes up protons, causing a decrease in the pH inside the endosome [31]. However, the polymer binds protons and prevents this pH decrease, causing a greater influx of protons and anions into the endosome and thereby enhancing the ionic power and water entry into the endosome. This process is termed a proton pump. The excess water entry causes the endosome to burst, releasing the polyplex into the cytosol [32,33]. In this way, an increase in the cationic density and buffer capacity can increase the chance that the polyplex will reach the cytosol. However, recent studies have shown that endosomal escape cannot be explained solely by the proton sponge effect and involves other complex mechanisms [34]. Another aspect of concern is DNA release from the polyplex, because DNA that is too strongly bound to the polyplex will show poor transfection efficiency. 

The mean pH inside the endosomes is pH 5–6, so the maximum buffer capacity of a CS derivative can be reached by introducing a substituent with a p*K*a in the range of 5–6 [35]. Compounds with this p*K*a include pyridine (p*K*a 5.2 [36]) and its derivatives, such as pyridoxal, which is one form of vitamin B_6_. Pyridoxal contains a pyridine ring and aldehyde group, making it a suitable low-toxicity [37] reagent for the chemical modification of CS. 

**Figure 1:**
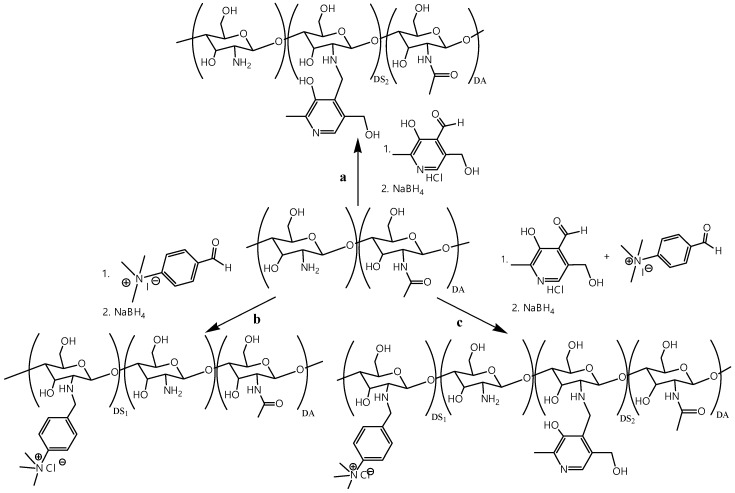
Synthesis scheme for *n*-[(3-hydroxy-5-(hydroxymethyl)-2-methyl-4-pyridine)methyl]chitosan chloride (Pyr-CS) (**a**), *n*-[4-(*n*’,*n*’,*n*’-trimethylammonium)benzyl]chitosan chloride (TMAB-CS) (**b**), and *n*-[4-(*n*’,*n*’,*n*’-trimethylammonium)benzyl]-*n*-[(3-hydroxy-5-(hydroxymethyl)-2-methyl-4-pyridine)methyl]chitosan chloride (PyrTMAB-CS) (**c**).

Previous studies [19] established that the transfection efficiency is higher with TMAB-CS with a relatively low DS of 0.25 than with highly substituted TMAB-CS due to better polyplex dissociation within the cell. All three CS derivatives were obtained by a two-step one-pot procedure (Figure 1). The reactions of CS and the corresponding aldehydes resulted in the formation of Schiff bases, which were subsequently reduced with NaBH_4_. The appearance of a yellow coloration indicated imine bond formation, while the reduction of the formed imine resulted in bleaching of the reaction mixture. After completion of the reduction, all three samples were precipitated with acetone, purified by dialysis, and freeze dried. The structures and DS of Pyr-CS, TMAB-CS, and PyrTMAB-CS were confirmed by ^1^H NMR spectroscopy. 

**Figure 2:**
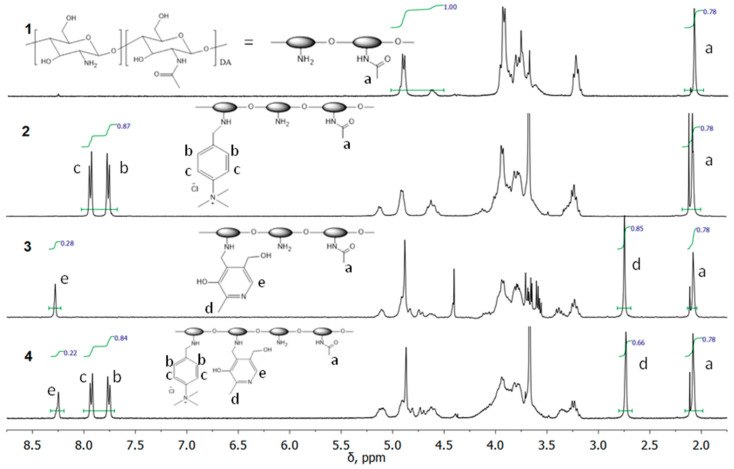
^1^H NMR spectra of CS (1), TMAB-CS (2), Pyr-CS (3), and PyrTMAB-CS (4).

The addition of the TMAB-moiety to the CS backbone was confirmed by ^1^H NMR (Figure 2), which showed the appearance of the signals of the aromatic protons (b, c) at around 7.85 ppm and the methyl protons at 3.67 ppm.

The addition of the pyridoxal substituent was confirmed by the appearance of the signals of the aromatic proton (e) at 8.25 ppm and methyl protons (d) at 2.75 ppm.

The addition of both substituents was confirmed by the appearance of the signals of the aromatic proton (e) at 8.25 ppm and methyl protons (d) at 2.75 ppm from the pyridine ring, as well as the signals of the aromatic protons (b, c) at around 7.85 ppm and the methyl protons at 3.67 ppm from TMAB-moiety.

The signal of acetamide protons (a) at 2.08 ppm (3 DA, 0.78 H) was used as a reference signal for integration. DS of the CS derivatives were calculated from the ^1^H NMR spectra as follows:

$$DS_{1}=\frac{I\left(b\right)}{2}=\frac{I\left(c\right)}{2},\;DS_{2}=I\left(e\right)=\frac{I\left(d\right)}{3}$$

The following results were obtained:

TMAB-CS; DS_1_ = 0.22

Pyr-CS; DS_2_ = 0.28

PyrTMAB-CS; DS_1_ = 0.21 and DS_2_ = 0.22.

### 3.2. Spectrophotometric pKa Determination

**Figure 3:**
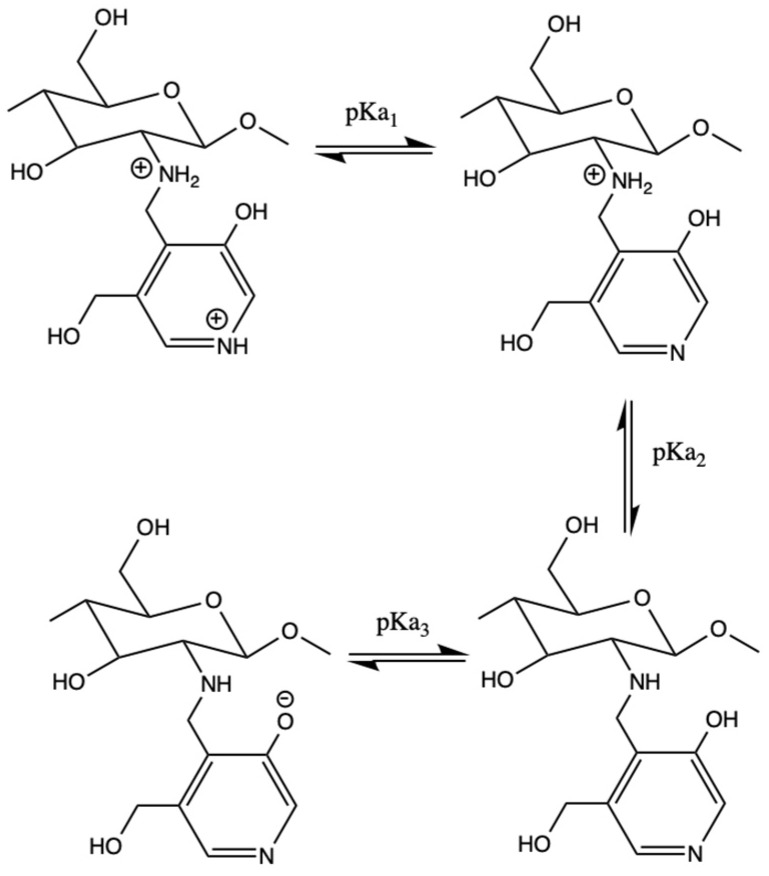
Ionization scheme of Pyr-CS units.

**Figure 4:**
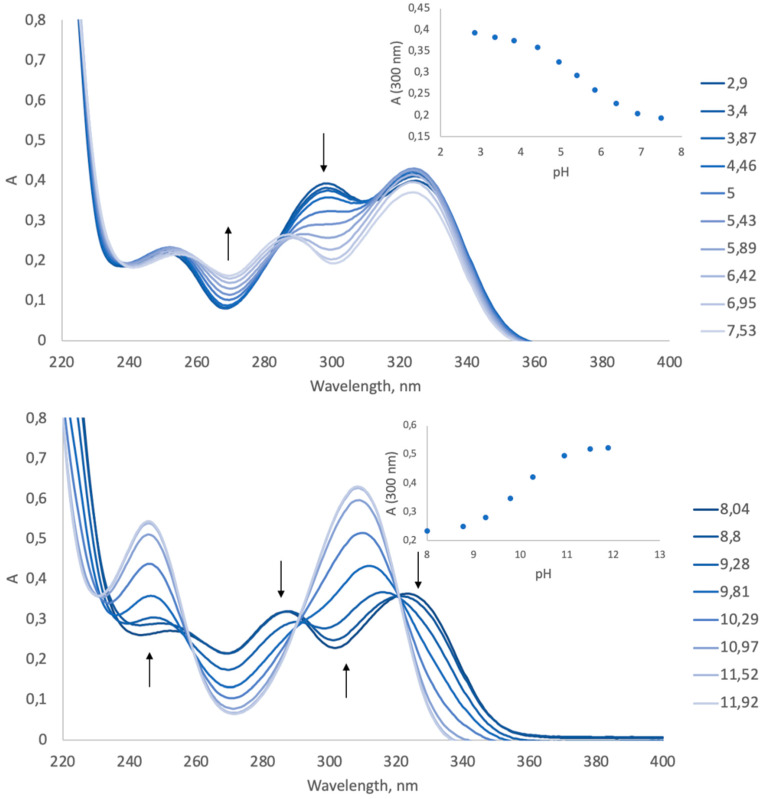
Evolution of the UV spectrum of Pyr-CS (0.125 mg/mL) with changing pH.

Maturation of the endosome leads to its internal acidification to a pH of about pH 5.0 (compared to pH 7.4 in the cytoplasm). Therefore, an efficient vector that works via the proton sponge effect should maintain the buffer capacity in that pH range. CS has its own amino groups with p*K*a ~6.4 [38]. The p*K*a values of the Pyr-substituent can be determined from UV-spectra, as the spectra are sensitive to ionization of the Pyr-substituent (Figure 3). We analyzed the UV-spectra to define the p*K*a values of the Pyr-substituent in Pyr-CS (Figure 4), PyrTMAB-CS, and pyridoxal. The p*K*a values calculated from the absorbance–pH dependences (insets in Figure 4) are summarized in Table 1. We were unable to determine the p*K*a_3_ of PyrTMAB-CS due to the flocculation of the polyampholyte in the pH range near the isoelectric point [39] caused by ionic interaction between the positively charged quaternary ammonium group of TMAB-substituent and the negatively charged phenolate ion of the Pyr-substituent. Both Pyr-CS and PyrTMAB-CS exhibit higher p*K*a_1_ values (5.5 and 4.9, respectively) than pyridoxal (Table 1). Since the highest buffer capacity is at a pH value equivalent to the p*K*a value, this might have a positive effect on the transfection efficiency due to the proton sponge *K*.

### 3.3. Preparation and Hydrodynamic Parameters of Polymer:DNA Polyplexes

The three polymers were each dissolved in PBS, purified with a sterilizing filter, and added by titration to plasmid DNA solution to reach various polymer:DNA mass ratios. After polyplex formation, the R_h_ of the polyplexes was measured by the DLS method.

**Figure 5:**
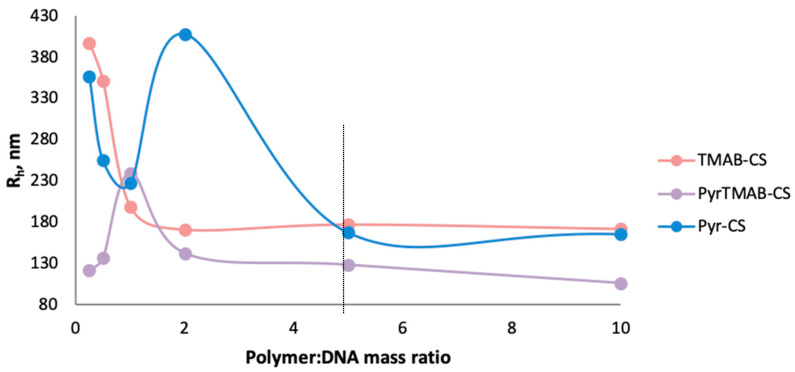
The hydrodynamic radii of TMAB-CS:DNA, Pyr-CS:DNA, and PyrTMAB-CS:DNA polyplexes.

Using of low concentrations of the polymers (polymer:DNA ratio 0.5:1 and 1:1) led to the formation of structures and aggregates of various sizes because of the excess of negatively charged DNA. At a polymer:DNA ratio of 2:1, the size of the Pyr-CS-based complexes significantly exceeded the sizes of the TMAB-CS:DNA and PyrTMAB-CS:DNA complexes due to weaker DNA binding with Pyr-CS than with other derivatives. An increase in the polymer concentration in all three cases led to a decrease in the hydrodynamic radius, which can be explained by an increase in the ionic interaction between the polymers and DNA, resulting in a more compact packing of the complexes. The size of polyplexes stabilized at a mass ratio of polymer: DNA 5:1 (Figure 5); therefore, with a fivefold excess of polymer, the average R_h_ was 177 nm for TMAB-CS, 168 nm for Pyr-CS, and 115 nm for PyrTMAB-CS.

### 3.4. DNA Binding Assay

Ethidium bromide is a fluorescent intercalating agent; therefore, the fluorescence intensity is significantly higher for the DNA-bound dye than for free ethidium bromide. When CS is added to a solution containing DNA and EtBr, the cationic polymer binds DNA and displaces EtBr, thereby quenching the fluorescence. Thus, the ability of polymers to bind DNA can be estimated by the fluorescence quenching pattern [40].

**Figure 6:**
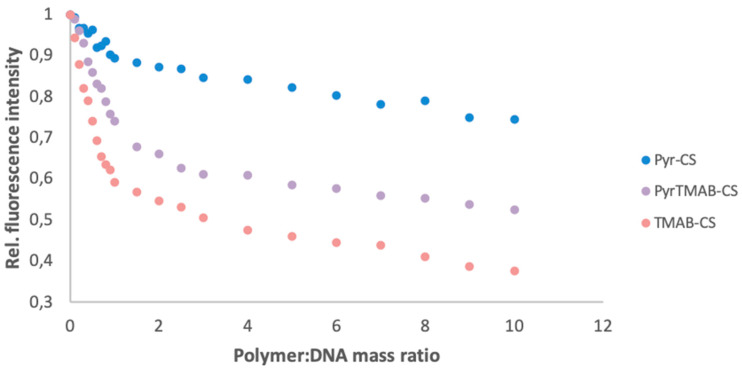
DNA binding ability for Pyr-CS, PyrTMAB-CS, and TMAB-CS as determined by the relative fluorescence changes.

The signal strength associated with EtBr DNA was used as a baseline value (SEtBr). Next, a solution of a CS derivative (0.1 to 10 eq) was added to the solution, and the fluorescence intensity was measured after each addition *(S_i_)*. The relative change in fluorescence (∆*i*) was calculated as the ratio of the change in fluorescence intensity before and after the addition of DNA *(S_i-1_ -S_i_)* versus the change in fluorescence intensity of the base solution and the final solution *(S_EtBr_-S_i_)*. As shown in Figure 6, TMAB-CS showed the greatest ability to bind DNA, while Pyr-CS showed the least ability. This result can be explained by a better bonding between the quaternary ammonium group and DNA. 

### 3.5. AFM Morphology of Polymer:DNA Polyplexes

**Figure 7:**
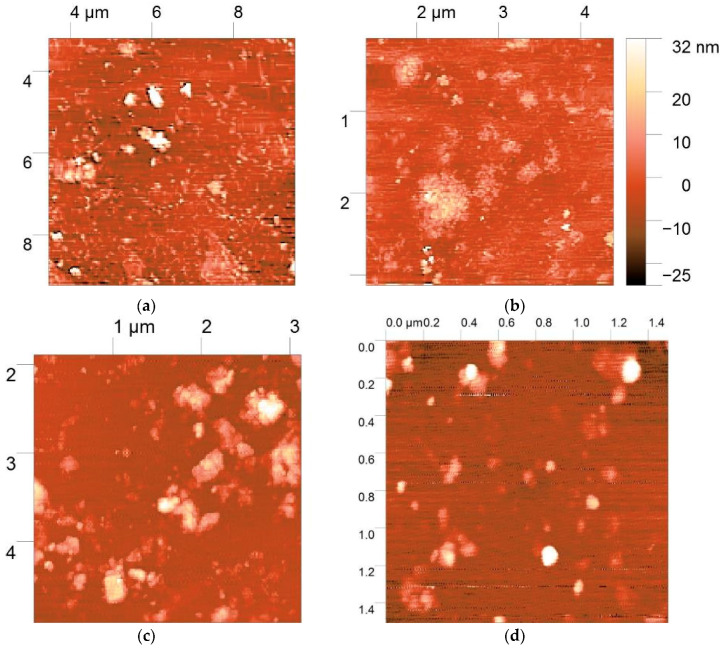
Atomic force microscopy (AFM) images of DNA-polyplexes with TMAB-CS (**a**), Pyr-CS (**b**), and PyrTMAB-CS (**c**,**d**).

The morphology and size distribution of the polyplexes prepared at the polymer:DNA ratio of 5:1 were studied by AFM. The polyplex particles formed by all three derivatives had elongated or nearly spherical forms of low-density texture (Figure 7a–c). The polyplex height was less than 30 nm, while the x- and y-sizes had relatively broad distributions in the range of 50–500 nm. The polyplexes were placed on the surface of negatively charged mica to allow interactions to occur between the positively charged polyplexes (the ζ-potential was approximately +22 mV at the ratio of 5:1) and the mica surface. These interactions resulted in spreading and partial unraveling of the polyplexes on the surface. Thus, the particle sizes of the polyplexes obtained by AFM correlated with the values obtained for the hydrodynamic radii at the ratio of 5:1. The discrepancy in the results can be explained by the differences in the measurement conditions, i.e., drying of the polyplexes before AFM measurement and their interaction with the oppositely charged mica surface.

Danielsen et al. [41] previously used AFM to examine a blend of toroid and rod forms with heights of 10–15 nm. The toroid-to-rod ratio decreased with increasing DA of CS, indicating an influence of the CS charge density on the formation of denser rod structures. Fuzzy, non-regular, spherical shapes were obtained for low molecular weight CS (4–22 × 10^3^); these polyplexes also had a higher transfection efficiency [26]. At high CS-to-DNA ratios (up to 60), the polyplex structures became also fuzzier due to the formation of additional polysaccharide shells [42]. Thus, our relatively low DP and high CS:DNA ratio led to formation of less dense polyplexes that could easily release DNA. Denser structures with shapes close to spheres or aggregates were observed for PyrTMAB-CS (Figure 7d), indicating a denser DNA packing and more stable complex formation. Similar structures were observed for poly(amido amine) [43] and polypeptide [44] polyplexes by real-time AFM in liquid. The release of DNA was promoted by partial destruction of the carrier into shorter fragments, as well as by passage through toroid formation, aggregation of the toroids into larger structures, and unraveling of DNA from the polyplex. This resulted in highly decondensed, wormlike chains and loops that were held by a central compact core.

### 3.6. Transfection of the HEK293 Cell Line

**Figure 8:**
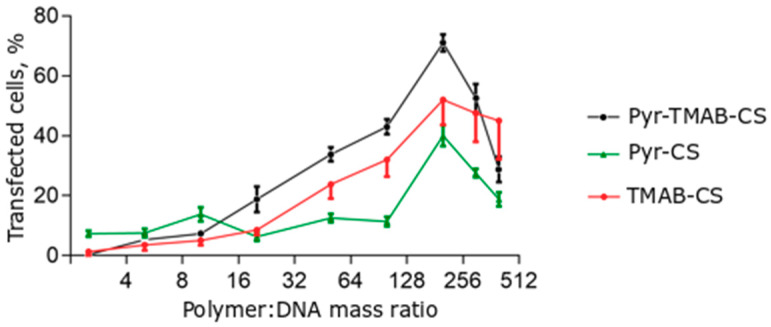
Transfection efficiency of polyplexes at different polymer:DNA mass ratios. Individual points represent mean ± SD, n = 8.

**Figure 9:**
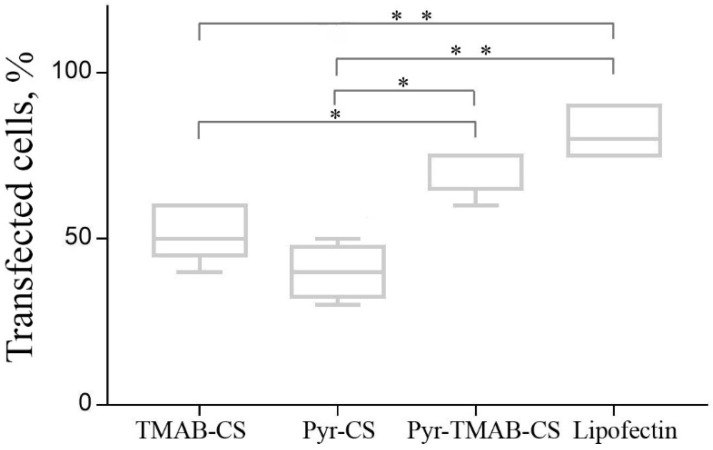
Transfection efficiency of polyplexes at 200:1 polymer:DNA mass ratio compared to that of Lipofectin. Each column represents the so-called “five-number summary” and describes a list of five values: the minimum, the 5th percentile, the median, the 95th percentile, and the maximum. Horizontal brackets with asterisks indicate statistically significant differences: * *p* < 0.01; ** *p* < 0.001.

The transfection efficiency of the polyplexes was investigated in the HEK293 cell line. The transfection efficiency assay was conducted using a ratio of CS derivatives to plasmid DNA that varied from 2.5:1 to 400:1 (Figure 8). The transfection efficiency was estimated by counting the numbers of cells expressing the green fluorescent protein. A large excess of cationic polymer was required to reach a sufficient transfection level, in agreement with previously published results. However, the efficiency relationship at certain ratios differed for different ranges. At low polymer:DNA ratios (2.5:1–10:1), Pyr-CS was more effective at transfection (although still less than 20% of the cells expressed GFP). With a cationic polymer excess higher than 10-fold, the vectors containing quaternary ammonium substituents were significantly more effective than Pyr-CS. This may reflect a promotion of transfection at high +/− ratios of the free polymer by neutralization of anionic glycosaminoglycans on the cell surface and by endosome membrane disruption. Both of these responses were more pronounced in the case of highly charged polymers. Maximum efficiency was reached at a mass ratio of 200:1 (50%–70%) and was slightly lower than that achieved with the commercial transfection agent Lipofectin (Figure 9). However, even the highest concentration of CS derivatives caused no toxic effects in the cell culture.

## 4. Conclusions

In this paper, three novel cationic CS derivatives containing either a quaternary ammonium group (TMAB-CS), pyridine (Pyr-CS), or both substituents (PyrTMAB-CS) were synthesized to evaluate the influence of the substituent structure on the transfection efficiency on the HEK293 cell line and to determine the presence, if any, of a synergistic effect. 

According to the experimental results, Pyr-CS demonstrated a higher transfection efficiency at low polymer:DNA ratios. However, when used at a greater excess, the Pyr-CS showed the least efficiency, while the mixed-substituted PyrTMAB-CS showed the best transfection efficiency, achieving a 70% transfection of the cells. Based on the results of the DNA binding assay, the TMAB-substituent has a greater effect on the binding of DNA to the polymer. Pyr-CS has a significantly lower ability to bind DNA, but it is able to function as a proton sponge due to presence of the pyridine moiety with a p*K*a ~5 that affects the DNA release from the polyplex. Thus, the introduction of two different substituents into CS allows the formation of a polymer vector that is able to bind to plasmid DNA due to its quaternary ammonium substituent and to release DNA in target cells due to its Pyr-substituent that functions as a proton sponge. The end result is an increase in the transfection efficiency of the CS-based vector.

## Figures and Tables

**Table 1:** The p*K*a values of Pyr-containing compounds.

Compound	p*K*a_1_	p*K*a_3_
Pyr-CS	5.5	10
PyrTMAB-CS	4.9	-
Pyridoxal	4.2	8.8
